# IL-6 Promotes Islet *β*-Cell Dysfunction in Rat Collagen-Induced Arthritis

**DOI:** 10.1155/2016/7592931

**Published:** 2016-11-14

**Authors:** Huan Jin, Yaogui Ning, Haotong Zhou, Youlian Wang

**Affiliations:** ^1^Department of Rheumatology, Jiangxi Provincial People's Hospital, Nanchang 330006, China; ^2^Department of Critical Care Medicine, The First Affiliated Hospital of Xiamen University, Xiamen 361003, China

## Abstract

The aim of this study was to explore the possible mechanism of rheumatoid arthritis- (RA-) related abnormal glucose metabolism. The model of collagen-induced arthritis (CIA) was established by intradermal injection of type II collagen into Wistar rats; complete Freund's adjuvant injections were used as the control group. Fasting plasma glucose (FBG) was measured by the glucose oxidase method. Fasting insulin (FIns) and the expressions of IL-6 were detected by ELISA. Islet caspase-3 was examined by immunohistochemistry. On day 17 after immunization, FBG of the CIA group showed an elevated FBG value compared with the control group. Meanwhile, the FIns of group CIA was lower when compared with the control group. Interestingly, the inflammatory cytokine IL-6 expression was significantly increased when compared with the control group. As expected, the abnormal glucose metabolism was accompanied by the increased IL-6 expression. Furthermore, in line with the upregulated IL-6 expression, the apoptosis related enzyme caspase-3 was also markedly increased. These data showed that the elevated FBG in CIA may be associated with the reduced FIns level secondary to the overapoptosis of pancreas islet cells induced by IL-6.

## 1. Introduction

Rheumatoid arthritis (RA) is a chronic disease characterized by synovitis and autoantibody such as rheumatoid factor (RF) and anti-cyclic citrullinated peptide antibody (anti-CCP) [1, 2]. Besides the destruction of cartilage and bone, RA also leads to system damage including cardiovascular, pulmonary, and psychological systems [3, 4]. Blockage of proinflammatory cytokines activity involved in the chronic inflammation of RA, such as tumor necrosis factor-alpha (TNF-*α*) and interleukin-6 (IL-6), was applied in clinical therapy [5]. IL-6 is a pleiotropic proinflammatory cytokine produced by synovial cells and endothelial cells [6, 7]. IL-6 favors T-cell activation, B-cell proliferation, and immunoglobulin secretion [8]. Moreover, IL-6 participates in the synthesis of acute phase proteins in the liver and proliferation and differentiation of hematopoietic precursor cells. Its overexpression can promote the occurrence of RA and other autoimmune diseases [9].

Previous studies have defined the relationship between inflammatory factors and insulin resistance [10–12]. Increased levels of serum inflammatory marker IL-6 have been found in patients with type 2 diabetes [12]. In addition, IL-6 treatment induces beta-cell apoptosis via STAT-3-mediated nitric oxide production [13–15]. Recent reports have shown that the prevalence of diabetes mellitus (DM) in patients with RA is about 15%–19%, which is significantly higher than that of the global DM [16, 17]. The fasting plasma glucose (FBG) in RA patients with type 2 diabetes mellitus (T2DM) is higher than that of T2DM patients, suggesting that the islet *β*-cell damage in RA patients complicated with T2DM is more serious [11], and these may be related to their long-term systemic inflammatory status [18, 19]. However, the mechanism of RA with *β*-cell damage is still uncertain.

In this study, the model of CIA was established to observe the expressions of FBG, FIns, IL-6, and islet caspase-3. As expected, the abnormal glucose metabolism was accompanied by the increased IL-6 expression. Furthermore, in line with increased IL-6 expression, the apoptosis related enzyme caspase-3 was also markedly increased. These data showed that the possible mechanism of RA complicated with abnormal glucose metabolism might result from the *β*-cell apoptosis induced by IL-6.

## 2. Materials and Methods

### 2.1. Experimental Animals and Reagents

Female Wistar rats, weighing 150–180 g, were purchased from Kaifu district, Changsha city, East Laboratory Animal Service Department, and were bred in a specific pathogen-free (SPF) environment. The rats were adaptively bred one week before experiment. All rats were maintained (20–24°C and 55–60% humidity) under a standard 12-hour light/dark cycle. IL-6 ELISA kits were purchased from RayBiotech Company (Norcross, USA); insulin ELISA kits were purchased from Wuhan Elabscience Biotechnology Co., Ltd. (Wuhan, China); bovine type II collagen (C7806) and complete Freund's adjuvant were purchased from Sigma Company (Shanghai, China); rabbit anti-rat caspase-3 was provided by Abcam Company (Cambridge, USA). Two-step immunohistochemical kits and diaminobenzidine (DAB) color liquid were purchased from Beijing Zhong Shan-Golden Bridge Biological Technology Co., Ltd. (Beijing, China).

### 2.2. Animal Models

Bovine type II collagen (10 mg) was dissolved in 5 mL 0.1 mol/L acetic acid solution, maintained at 4°C overnight, and set aside. An equal dose of collagen solution and complete Freund's adjuvant was thoroughly mixed and completely emulsified; then, a CII/IFA emulsion (1 mg/mL) was freshly manufactured. Rats were injected with emulsion at the base of the tail and back within three points (day 1); animals were boosted with the dose of 0.2 mL CII-IFA emulsion at day 7 by the same method. The control group were treated with complete Freund's adjuvant according to the same protocol. After initial immunization [20], the rats' arthritis index (AI) was scored as in a previous report. Blood samples were collected from the intraocular adjoined veins of the rats on the 17th day after initial immunization.

### 2.3. Immunohistochemistry

The pancreatic tissue was fixed in 4% paraformaldehyde (pH 7.4) for histological analysis. To detect the expression of caspase-3, the slides were incubated with antibody diluted at 1 : 80 overnight at 4°C, followed by DAB coloration, and stained with hematoxylin dye and dehydrated (transparent). Image-Pro Plus 6.0 was used to analyze and carefully delineate the scope of each field in the islets, to measure the mean absorbance of the islets, as an index for expression of islets caspase-3.

### 2.4. Immunoblots

 The protein extracted from the pancreatic tissue was assessed using an immunoblot assay. Antibodies against cleaved caspase-3 were obtained from Cell Signaling Technology (MA, USA). After incubating with secondary antibody conjugated horseradish peroxidase, the immunoreactivity was developed by an ECL system (Pierce).

### 2.5. Statistical Analysis

All statistical analyses were performed using Prism 5 software. Data are presented as the mean ± standard error (SD). Group comparisons were performed using Student's *t*-test. Paired Student's *t*-test was used for paired data. A value of *p* < 0.05 was considered as statistically significant.

## 3. Results

### 3.1. Abnormal Glucose Metabolism in Rat Collagen-Induced Arthritis

**Figure 1:**
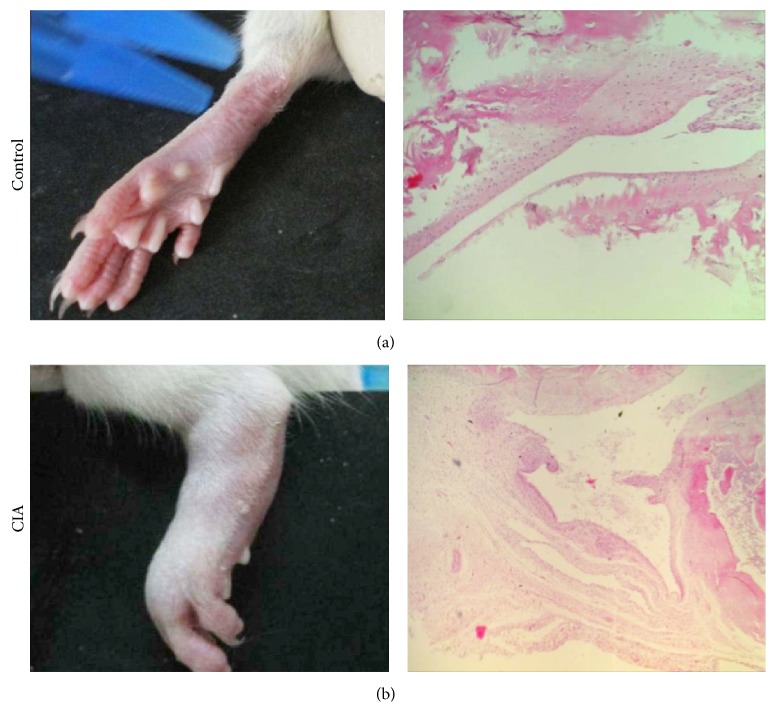
Establishment of rat collagen-induced arthritis. After day 17 of immunization, the macroscopic change of hind limbs and the histology of joint tissue sections from CIA rats and control rats were analyzed. All data are representative of one out of three independent experiments.

**Figure 2:**
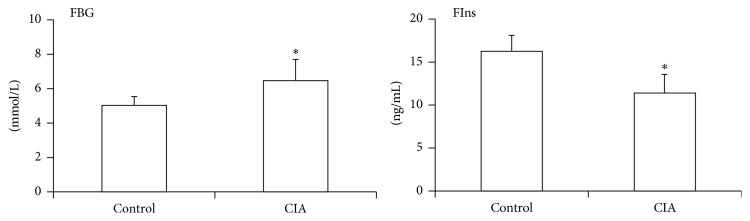
Glucose metabolism dysfunction in CIA rats. Blood samples were collected from the intraocular adjoined veins of the rats on day 17 after initial immunization from CIA rats and control rats. FBG and FIns in sera of both groups were determined as described in the Materials and Methods. The data are presented as mean ± SD (*n* = 6–8/group) and are representative of three independent experiments. ^*∗*^ *p* < 0.05.

In recent studies, there has been increased evidence of the relationship between glucose metabolism dysfunction and human autoimmune disease. To explore the mechanism of this observation in rheumatoid arthritis, rat collagen-induced arthritis model was established. In this study, on day 17 after the primary immunization, the CIA rats showed mild edema in one or both sides of the hind limbs. Furthermore, histological analysis showed hyperplasia of the synovial membrane and the infiltration of inflammatory cells in CIA rats (Figures 1(a) and 1(b)). As expected, a markedly increased level of FBG was observed in CIA rats when compared with the control group. Meanwhile, a decreased level of FIns was observed in CIA rats when compared with the control group (Figure 2). These data suggest abnormal glucose metabolism in rat collagen-induced arthritis.

### 3.2. Effect of Increased IL-6 Expression on FBG and FIns

**Figure 3:**
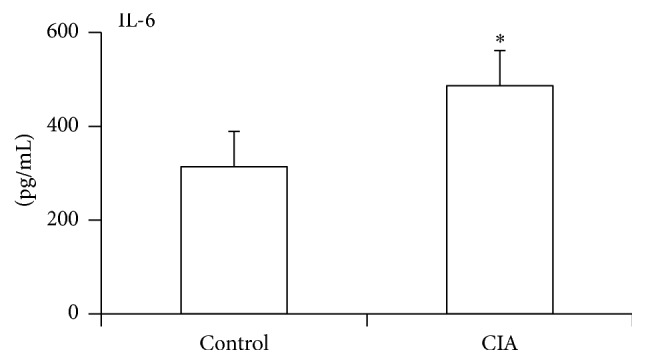
Increased IL-6 expression in rat collagen-induced arthritis. Blood samples were collected from the intraocular adjoined veins of the rats on day 17 after initial immunization from CIA rats and control rats. IL-6 in sera was determined by ELISA kit. The data are presented as mean ± SD (*n* = 6–8/group) and are representative of three independent experiments. ^*∗*^ *p* < 0.05.

**Figure 4:**
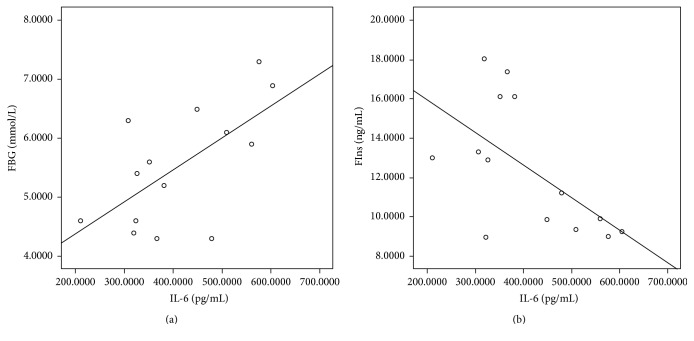
Correlation between IL-6 and FBG and FIns. The sera were harvested after day 17 of immunization. Levels of IL-6, FBG, and FIns were determined. The correlations between IL-6 and these factors in all the rats were analyzed by Spearman correlation test. *p* value < 0.05 was considered as statistically significant. Data are representative of three independent experiments.

Previous studies have defined the relationship between inflammatory cytokines and insulin resistance [15]. IL-6 is a critical inflammatory cytokine in the pathogenesis of RA, and blockage of its activity by IL-6R antibody was applied in clinical treatment. Here, we also observed an increased expression of IL-6 in CIA rats when compared with control (Figure 3). An increased IL-6 level was also observed in patients with type 2 diabetes. Next, to determine the role of IL-6 in the regulation of glucose metabolism in CIA rats, the correlation between IL-6 and FBG or FIns was analyzed. The result met our expectations; a significantly positive correlation was observed between IL-6 and FBG (Figure 4(a)). In addition, a negative correlation was depicted in IL-6 and FIns (Figure 4(b)).

### 3.3. IL-6 Promotes Caspase-3 Expression in Pancreas Islet

**Figure 5:**
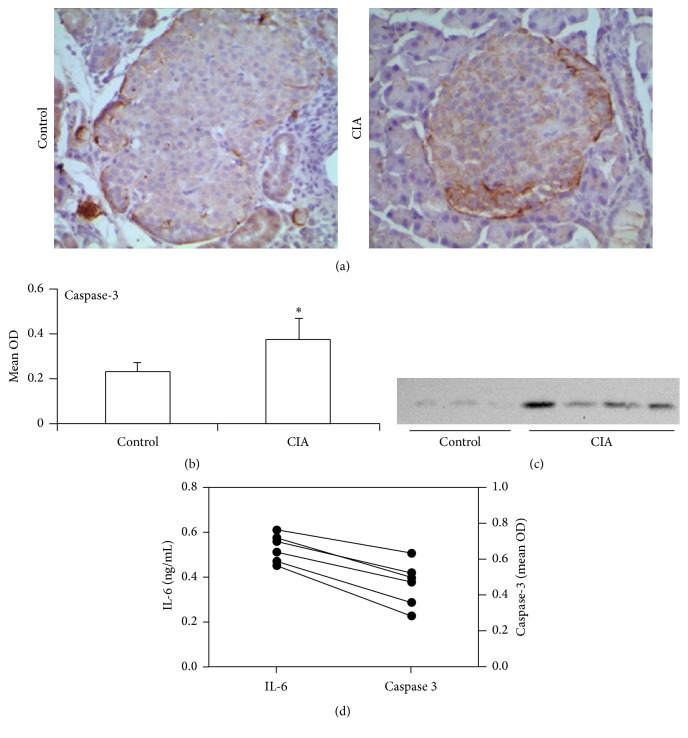
Potential of IL-6 in the apoptosis of *β*-cells. The pancreas islets were harvested after day 17 of immunization. (a) The apoptosis related gene caspase-3 was analyzed by immunohistochemistry. (b) The expression of caspase-3 was analyzed by OD value. Image-Pro Plus 6.0 was used to delineate the scope of each field in the islets and measure the mean absorbance of the islets. (c) The proteins of pancreas islets were extracted for analyzing cleaved caspase-3 expression by immunoblots assays. Data are representative of three independent experiments (d) Paired Student's *t*-test was used for analyzing the paired data of IL-6 and caspase-3. Data are shown as the mean ± SD (*n* = 6–8/group) and are representative of three independent experiments. ^*∗*^ *p* < 0.05.

A critical role of *β*-cell apoptosis has been demonstrated in the development of diabetes. In line with an increased expression of IL-6 (Figure 3), the apoptosis related enzyme caspase-3 was also markedly increased in CIA rats (Figures 5(a)–5(c)). Furthermore, the cleaved caspase-3 was also detected, as expected, and the CIA rats showed an increased level when compared with control rats. Previous studies have shown that IL-6 treatment induces beta-cell apoptosis via STAT-3-mediated nitric oxide production. Accordingly, a positive correlation between caspase-3 and IL-6 was observed in our study. Taken together, these results suggest that the abnormal glucose metabolism might be due to the *β*-cell apoptosis induced by inflammatory cytokine IL-6 (Figure 5(d)).

## 4. Discussion

Recent studies have shown that the prevalence of RA patients with abnormal glucose metabolism was significantly increased [11, 16], and the decreased insulin sensitivity and islet *β*-cell function due to islet *β*-cell excessive apoptosis in these patients and the mechanism might be related to overexpressed proinflammatory cytokines [21]. In this experiment, the expression of IL-6 in group CIA was significantly higher than that of control group, and the abnormal glucose metabolism was accompanied by the increased IL-6 expression. Furthermore, in line with increased IL-6 expression, the apoptosis related enzyme caspase-3 was also markedly increased. These data showed that the abnormal glucose metabolism in RA might result from the *β*-cell apoptosis induced by IL-6.

IL-6, an important proinflammatory cytokine, could promote insulin secretion at low concentration of IL-6 [22]. Interestingly, the high concentration of IL-6 would damage *β*-cells and promote apoptosis [13]. The cytotoxicity of high IL-6 concentrations could upregulate the expression of cleaved caspase-3 and phosphorylated p38 mitogen-activated protein kinase and phosphorylated nuclear factor-*κ*B, in part via signal transducers and activators of transcription-3-mediated NO production [8]. In addition, IL-6 can increase lipolysis in adipocytes as well as the release of free fatty acids, thereby damaging the mitochondria and glucose transporter 2 (GLUT2) function and insulin sensitivity [23]. Here, in our study, we also observed elevated IL-6 expression in CIA, which positively correlated with glucose. Furthermore, IL-6 can also induce the production of IL-1*β*, IL-2, and TNF-*α* and enhance the biological effects when combined with these cytokines [9]. The previous studies have also found that TNF-*α* produced by adipose tissue can induce insulin resistance (IR); these data showed the relationship between inflammatory factors and insulin resistance. Not only were IL-6 levels in CIA rats significantly increased, but also other proinflammatory cytokines like TNF-*α* and IFN-*γ* were overexpressed [5]. IL-6 can also be combined with TNF-*α*, IL-1*β*, and induced classical caspase-dependent apoptosis pathway, thereby increasing the apoptosis of *β*-cells and causing a reduction of insulin secretion [14]. These might be the reason for the high prevalence of diabetes mellitus (DM) in patients with RA when compared with healthy controls.

Insulin is one of the most important hormones in an organism that regulates blood sugar levels, which is secreted by pancreatic *β*-cells; insulin deficiency can lead to elevated blood glucose levels [21]. As expected, we observed that fasting serum insulin levels were significantly decreased in CIA. Furthermore, the increased expression of islet caspase-3 was also detected. Caspase-3 is an important proapoptotic protein and is the performer of cell apoptosis. When the cells are stimulated by the proapoptotic factors, caspase-3 can be activated and then induce their apoptosis [24, 25]. In addition, IL-6 was positively correlated with caspase-3 production which leads *β*-cell to apoptosis in our study. The elevated levels of IL-6 inhibiting the insulin secretion of islet *β*-cells might be through promoting *β*-cells apoptosis.

This study suggests the significantly increased FBG and decreased FIns in CIA rats, which might be a result from autoimmune inflammatory mediators such as IL-6. IL-6 induced abnormal glucose metabolism might be through upregulating caspase-3 expression, because of the positive correlation between IL-6 and apoptosis related enzyme caspase-3. Taken together, these data showed that the elevated FBG and reduced FIns level in CIA may be induced by the inflammatory cytokines generated by arthritis, which is significantly different from the traditional T2DM. The treatment of the primary disease in RA combined with DM can improve glucose metabolism.

## References

[1] Kourilovitch M., Galarza-Maldonado C., Ortiz-Prado E. (2014). Diagnosis and classification of rheumatoid arthritis. *Journal of Autoimmunity*.

[2] Cassidy J. T., Levinson J. E., Bass J. C. (1986). A study of classification criteria for a diagnosis of juvenile rheumatoid arthritis. *Arthritis and Rheumatism*.

[3] Maradit-Kremers H., Nicola P. J., Crowson C. S., Ballman K. V., Gabriel S. E. (2005). Cardiovascular death in rheumatoid arthritis: a population-based study. *Arthritis & Rheumatism*.

[4] Wagan A. A., Mahmud T. H., Rasheed A., Zafar Z. A., Rehman A. U., Ali A. (2016). Cardiovascular risk score in Rheumatoid Arthritis. *Pakistan Journal of Medical Sciences*.

[5] McInnes I. B., Buckley C. D., Isaacs J. D. (2016). Cytokines in rheumatoid arthritis-shaping the immunological landscape. *Nature Reviews Rheumatology*.

[6] Koga T., Yamasaki S., Migita K. (2011). Post-transcriptional regulation of IL-6 production by Zc3h12a in fibroblast-like synovial cells. *Clinical and Experimental Rheumatology*.

[7] Mihara M., Moriya Y., Kishimoto T., Ohsugi Y. (1995). Interleukin-6 (IL-6) induces the proliferation of synovial fibroblastic cells in the presence of soluble IL-6 receptor. *British Journal of Rheumatology*.

[8] Neurath M. F., Finotto S. (2011). IL-6 signaling in autoimmunity, chronic inflammation and inflammation-associated cancer. *Cytokine and Growth Factor Reviews*.

[9] Calabrese L. H., Rose-John S. (2014). IL-6 biology: implications for clinical targeting in rheumatic disease. *Nature Reviews Rheumatology*.

[10] Manrique-Arija S., Ureña I., Valdivielso P. (2016). Insulin resistance and levels of adipokines in patients with untreated early rheumatoid arthritis. *Clinical Rheumatology*.

[11] Shahin D., Eltoraby E., Mesbah A., Houssen M. (2010). Insulin resistance in early untreated rheumatoid arthritis patients. *Clinical Biochemistry*.

[12] Fève B., Bastard J.-P. (2009). The role of interleukins in insulin resistance and type 2 diabetes mellitus. *Nature Reviews Endocrinology*.

[13] Oh Y. S., Lee Y.-J., Park E. Y., Jun H.-S. (2011). Interleukin-6 treatment induces beta-cell apoptosis via STAT-3-mediated nitric oxide production. *Diabetes/Metabolism Research and Reviews*.

[14] Kim S., Kim K., Suk K., Kim Y., Oh S. H., Lee M. (2012). Apoptosis of human islet cells by cytokines. *Immune Network*.

[15] Delaney C. A., Pavlovic D., Hoorens A., Pipeleers D. G., Eizirik D. L. (1997). Cytokines induce deoxyribonucleic acid strand breaks and apoptosis in human pancreatic islet cells. *Endocrinology*.

[16] Simard J. F., Mittleman M. A. (2007). Prevalent rheumatoid arthritis and diabetes among NHANES III participants aged 60 and older. *The Journal of Rheumatology*.

[17] Pamuk Ö. N., Ünlü E., Çakir N. (2006). Role of insulin resistance in increased frequency of atherosclerosis detected by carotid ultrasonography in rheumatoid arthritis. *Journal of Rheumatology*.

[18] Mavrogeni S., Dimitroulas T., Bucciarelli-Ducci C. (2014). Rheumatoid arthritis: an autoimmune disease with female preponderance and cardiovascular risk equivalent to diabetes mellitus: Role of cardiovascular magnetic resonance. *Inflammation and Allergy-Drug Targets*.

[19] Bartels C. M., Saucier J. M., Thorpe C. T. (2012). Monitoring diabetes in patients with and without rheumatoid arthritis: A Medicare Study. *Arthritis Research and Therapy*.

[20] Salvemini D., Mazzon E., Dugo L. (2001). Amelioration of joint disease in a rat model of collagen-induced arthritis by M40403, a superoxide dismutase mimetic. *Arthritis & Rheumatism*.

[21] Khodabandehloo H., Gorgani-Firuzjaee S., Panahi G., Meshkani R. (2016). Molecular and cellular mechanisms linking inflammation to insulin resistance and *β*-cell dysfunction. *Translational Research*.

[22] Osório J. (2015). Diabetes: IL-6 mediates the protective effects of exercise on *β* cells. *Nature Reviews Endocrinology*.

[23] Dessein P. H., Joffe B. I., Stanwix A. E. (2002). Effects of disease modifying agents and dietary intervention on insulin resistance and dyslipidemia in inflammatory arthritis: a pilot study. *Arthritis Research*.

[24] Augstein P., Bahr J., Wachlin G. (2004). Cytokines activate caspase-3 in insulinoma cells of diabetes-prone NOD mice directly and via upregulation of Fas. *Journal of Autoimmunity*.

[25] Reddy S., Bradley J., Ross J. M. (2003). Immunolocalization of caspase-3 in pancreatic islets of NOD mice during cyclophosphamide-accelerated diabetes. *Annals of the New York Academy of Sciences*.

